# Children of parents with depression or anxiety: Long‐term follow‐up, causality and resilience

**DOI:** 10.1002/jcv2.12211

**Published:** 2023-11-28

**Authors:** Henrik Larsson

**Affiliations:** ^1^ School of Medical Sciences Örebro University Örebro Sweden

**Keywords:** causation, child development, epidemiology, longitudinal research, parental depression, resilience

## Abstract

Three papers in the December issue (2023) of *JCPP Advances* focus on children of parents with depression or anxiety. They highlight the value of using prospective longitudinal data to improve the understanding about the development of children of parents with depression or anxiety from early childhood to young adulthood. They contribute to an advanced understanding of long‐term outcomes, causality and resilience for children of parents with depression or anxiety.

Three papers in the December issue (2023) of *JCPP Advances* focus on children of parents with depression or anxiety. Parental depression and anxiety are both highly prevalent psychiatric conditions, representing a major public health concern. The aim of this editorial is to provide context to the findings of these three studies and to highlight important methodological strengths.

## FINDINGS IN CONTEXT

Narrative and systematic reviews (Downey & Coyne, 1990; Goodman & Gotlib, 1999; Lawrence et al., 2019) have during the past three decades, summarised the evidence around the extent to which parental depression or anxiety influence childhood outcomes, and why. The three papers in the current issue of JCPP Advances make important contributions to this longstanding research direction. The three studies are all based on internationally recognised and ongoing data collections using prospective longitudinal designs; Powell et al. (2023) used data from the Early Prediction of Adolescent Depression (EPAD) study, Ahmadzadeh et al. (2023) used the Norwegian Mother, Father and Child Cohort Study (MoBa), while Maruyama et al. (2023) used information from the 2004 Pelotas Birth Cohort. EPAD is a longitudinal high‐risk study, whereas MoBa and the 2004 Pelotas are large, population‐based, birth cohort studies.

Among many things, the three studies showcase the critical value of prospective longitudinal data for developmental research and provide valuable insights into long‐term outcomes for children, the extent to which observed associations are consistent with a causal hypothesis and the role of protective factor (e.g., resilience).

### Long‐term follow‐up

Despite extensive research on the topic, few studies have examined the development of depression and anxiety in children of depressed parents as they transition to adulthood. The lack of studies is probably due to the substantial investment of effort needed to collect multiple waves of detailed data from a large number of individuals over extended periods of time. One important exception is the Yale cohort recruited in the early 1980s, which has tracked high‐risk offspring from ages 6 to 23 over 30 years. This seminal study (Weissman et al., 2016) has shown consistently high rates of psychopathology and poor social functioning among the children of depressed parents.

In the context of the important prior work by Weissman and team, the publication by Powell et al. (2023) in the current issue of JCPP Advances investigated the development of mood and anxiety disorders in children of depressed parents during the important transition from childhood to adulthood. The study followed 337 young people aged 9–17 years at baseline for 13 years. The findings revealed that a significant proportion of the participants experienced mood and anxiety disorders, with increasing prevalence rates from childhood to early adulthood. Notably, the transition from adolescence to adulthood marked a substantial rise in prevalence, especially in males. The study also found that a significant proportion of the participants reported functional impairment, harmful alcohol use and self‐harm or suicide attempts in early adulthood. These results underscore the need for prolonged vigilance and targeted interventions for children of depressed parents, especially during the transition to early adulthood.

### Causality

As highlighted above, parental depression or anxiety is strongly associated with poor child outcomes, but it remains unclear whether these associations reflects causation or genetic confounding. Causal pathways are present if parental depression or anxiety influence the child outcomes for example, via parenting practices. However, most (in not all) psychiatric conditions, behaviours and temperamental traits are under genetic influence (Plomin, 2022). This genetic influence comprises the effects of many genetic variants, each of which associate with many different psychiatric conditions, behaviours and temperamental traits (Plomin, 2022). This means that associations of parental depression or anxiety with child outcomes may at least partly be influenced by the same genetic variants, which the parent and child share by being related. In other words, associations between parental depression or anxiety and child outcomes may reflect genetic confounding. To better understand role of the causality underpinning parent–child associations, researchers can use extended family designs to adjust for genetic confounding.

Several influential reviews (McAdams et al., 2018) have illustrated how extended family designs based on samples of twins, siblings or cousins (or a combination) and their offspring can used to test hypotheses regarding the nature of intergenerational transmission and ask whether parent–child associations remain after accounting for their genetic relatedness. Briefly, the children of monozygotic twins are as related to their parent's co‐twin as they are to their own parent (they share 50% of their DNA). In contrast, the children of dizygotic twins share 25% of their genetic variance with their parent's co‐twin. Children share 25%, 12.5% and 6.25% of their genetic variance with their parent's sibling, half‐sibling and cousin, respectively. A large number of studies have already used extended family designs to better understand causality and the role of genetic confounding (McAdams et al., 2018). A major limitation of the prior extended family design studies is that most are based on cross‐sectional designs, which constrains possibilities to identify developmental processes.

The publication by Ahmadzadeh et al. (2023) in the current issue of JCPP Advances analysed longitudinal data together with an extended family design (or more specifically a multiple‐children‐of‐twins/siblings model). The longitudinal information on mothers' emotional symptoms (when children were aged 1.5, 3 and 5 years) and child temperament allowed the researchers to explore co‐development across generation, which is a novel component of this study. Ahmadzadeh et al. (2023) revealed that longitudinal stability in mothers' emotional symptoms aligned with stability in offspring emotionality, shyness and sociability. In contrast, the associations between changes in maternal symptoms and changes in child temperament were small or negligible. The study concluded that the observed associations between maternal emotional symptoms and child traits across time were primarily influenced by stable, trait‐like factors encompassing both genetic and environmental influences. These findings contribute valuable insights into the nuanced development of emotional symptoms within families and underscore the necessity of considering both genetic and environmental factors in understanding these associations over time.

### Resilience

Over the last three decades, there has been an increased interest from researchers, clinicians and policymakers in resilience (Rutter, 2012); probably because a comprehensive understanding of how resilience develops is crucial for identifying intervention targets to improve child outcomes related to adversity. Resilience research build on the universal observation of heterogeneity in outcomes following various adversities (Rutter, 2012). That is, not all children exposed to adversity develop poor outcomes, emphasizing the concept of resilience. There are several definitions of resilience and the approach used to study resilience differs across studies, in part depending on the research question (Cosco et al., 2017). One frequently used approach in prior resilience research, the person‐centred method, compares individuals exposed to similar adversities to distinguish those who remain well from those who develop mental health problems (Cosco et al., 2017).

Research using the person‐centred method has identified factors at various levels associated with resilience to parental depression or anxiety, including socioeconomic, family and individual factors (Collishaw et al., 2016). A critical limitation of this previous research is that many studies have examined these factors individually, overlooking their interrelatedness, potentially leading to inconsistent conclusions regarding the role of specific resilience factors. An even more important limitation is that prior studies have relied on cross‐sectional designs, which limits possibilities to investigate mediating processes.

The publication by Maruyama et al. (2023) in the current issue of JCPP Advances makes an important contribution to the field by addressing these limitations. The prospective longitudinal data allowed the researchers to focus on direct and indirect (i.e., mediation) pathways leading to child resilience in those exposed to maternal depression. Despite the negative impact of maternal depression on child mental health, a minority of children (12.4%) displayed resilience. Results from the sophisticated mediation analyses revealed indirect pathways from socioeconomic status to resilience through cognitive stimulation and intelligence quotient (IQ), indicating that higher socioeconomic status was associated with resilience through elevated cognitive stimulation and IQ levels. Notably, cognitive stimulation was identified as a direct and modifiable protective factor for children exposed to maternal depression, even after controlling for socioeconomic status. These findings underscore the significance of early childhood cognitive stimulation as a potential intervention target to promote child resilience in the context of maternal depression.

## CONCLUSIONS

An important focus for JCPP Advances is to continue to showcase the very best research from studies using sophisticated statistical analyses of data from longitudinal prospective studies combined with thoughtful study designs. The three papers in the current issue of JCPP Advances represent great examples of such research. The studies have highlighted the value of using prospective longitudinal data to improve the understanding about the development of children of parents with depression or anxiety from early childhood to young adulthood. They have more specifically contributed to an advanced understanding of long‐term outcomes, causality and resilience for children of parents with depression or anxiety.

## AUTHOR CONTRIBUTIONS

**Henrik Larsson**: Writing – original draft; writing – review & editing.

## CONFLICT OF INTEREST STATEMENT

Henrik Larsson reports receiving grants from Shire Pharmaceuticals; personal fees from and serving as a speaker for Shire Pharmaceuticals and Evolan Pharma AB outside the submitted work; and sponsorship for a conference on attention‐deficit/hyperactivity disorder from Shire Pharmaceuticals outside the submitted work. Henrik Larsson is Editor‐in‐chief of JCPP Advances. The author has declared that he has no competing or potential conflicts of interest with regards to this editorial.
